# Retraction Note: Effects of CREB1 gene silencing on cognitive dysfunction by mediating PKA-CREB signaling pathway in mice with vascular dementia

**DOI:** 10.1186/s10020-023-00708-4

**Published:** 2023-08-07

**Authors:** Xin-Rui Han, Xin Wen, Yong-Jian Wang, Shan Wang, Min Shen, Zi-Feng Zhang, Shao-Hua Fan, Qun Shan, Liang Wang, Meng-Qiu Li, Bin Hu, Chun-Hui Sun, Dong-Mei Wu, Jun Lu, Yuan-Lin Zheng

**Affiliations:** 1 Key Laboratory for Biotechnology on Medicinal Plants of Jiangsu Province, School of Life Science, Jiangsu Normal University, No. 101, Shanghai Road, Tongshan District, Xuzhou, Jiangsu Province 221116 People’s Republic of China; 2 College of Health Sciences, Jiangsu Normal University, No. 101, Shanghai Road, Tongshan District, Xuzhou, Jiangsu Province 221116 People’s Republic of China

Retraction to: Molecular Medicine (2018) 24:18 10.1186/s10020-018-0020-y

The Editor in Chief has retracted this article after an investigation by the Academic Committee of Jiangsu Normal University found image irregularities in Figs. 3 and 4 C, 6 A and 7 C. The Academic Committee of Jiangsu Normal University requested the original experimental records and data for verification from the authors of this article, however, the authors were unable to provide this. The Editor in Chief has therefore lost confidence in the integrity of the data and results of this article.

None of the authors responded to any correspondence from the editor about this retraction.

